# Evolving trends in infective endocarditis in a developing country: a consequence of medical progress?

**DOI:** 10.1186/s12941-019-0341-x

**Published:** 2019-12-17

**Authors:** Mohamed Sunil, Huynh Quoc Hieu, Ramesh Singh Arjan Singh, Sasheela Ponnampalavanar, Kelvin S. W. Siew, Alexander Loch

**Affiliations:** 1 Department of Medicine, University Malaya Medical Centre, 59100 Kuala Lumpur, Malaysia; 2 Tam Duc Cardiology Hospital, Ho Chi Minh City, Vietnam

**Keywords:** Infective endocarditis, *Staphylococcus aureus*, Healthcare associated infection, Mortality predictors, Malaysia

## Abstract

**Background:**

Staphylococcus has replaced streptococcus as the most common cause of infective endocarditis (IE) in developed health care systems. The trend in developing countries is less clear.

**Aim:**

To examine the epidemiological trends of infective endocarditis in a developing nation.

**Methods:**

Single-centre, retrospective study of patients admitted with IE to a tertiary hospital in Malaysia over a 12-year period.

**Results:**

The analysis included 182 patients (n = 153 Duke’s definite IE, n = 29 possible IE). The mean age was 51 years. Rheumatic heart disease was present in 42%, while 7.6% were immunocompromised. IE affected native valves in 171 (94%) cases. Health-care associated IE (HCAIE) was recorded in 68 (37.4%). IE admission rates increased from 25/100,000 admissions (2012) to 59/100,000 admissions (2017). At least one major complication on admission was detected in 59 (32.4%) patients. Left-sided IE was more common than right-sided IE [n = 159 (87.4%) vs. n = 18 (9.9%)]. Pathogens identified by blood culture were staphylococcus group [n = 58 (40.8%)], streptococcus group [n = 51 (35.9%)] and *Enterococcus* species [n = 13 (9.2%)]. staphylococcus infection was highest in the HCAIE group. In-hospital death occurred in 65 (35.7%) patients. In-hospital surgery was performed for 36 (19.8%) patients. At least one complication was documented in 163 (85.7%).

**Conclusion:**

Staphylococcus is the new etiologic champion, reflecting the transition of the healthcare system. Streptococcus is still an important culprit organism. The incidence rate of IE appears to be increasing. The rate of patients with underlying rheumatic heart disease is still high.

## Introduction

Infective endocarditis (IE) is a universal disease with risk factors, predispositions and outcomes considered similar across continents for decades. An epidemiological transition has been reported over the past decade for developed health care systems where viridans streptococcus has been replaced by staphylococcus as the most common etiological agent. Methicillin-susceptible *Staphylococcus aureus* (MSSA) is now the most common agent in native valve infective endocarditis (NVIE) and methicillin-resistant *S. aureus* (MRSA) in healthcare-associated infective endocarditis (HCAIE) [1]. This transition is thought to be a result of ageing populations, the abolition of rheumatic heart disease (RHD) as the traditional major risk factor for IE due to appropriate antibiotic treatment of rheumatic fever and advanced device management particularly in cardiac patients [2, 3]. Moreover, reductions in IE mortality rates that had been anticipated with improved medical care did not materialize due to the changing epidemiology and the coinciding higher rates of HCAIE [1, 4, 5]. It is not clear whether developing countries undergo an epidemiological transition of IE to the same extent. Available studies show notable differences in developing countries with much higher rates of culture-negative IE, zoonotic infections and mortality, although with significant regional variations [6, 7]. There is a lack of data for developing countries overall and South-East Asia in particular. The aim of this study is to describe the clinical trends, characteristics and outcomes of IE patients in a Malaysian tertiary hospital.

## Materials and methods

The study is a single-centre, retrospective study of patients admitted with IE to a tertiary hospital in Kuala Lumpur, Malaysia from 1st January 2005, until 31st December 2017. The hospital caters a population of 1.7 million with an annual adult admission rate of 44,000–46,000/year. The study received approval from the institutional ethics committee (No: 201782-5459).

### Patient recruitment and inclusion criteria

Patients were identified from the echocardiography reporting system, the admission logbooks and the hospital electronic medical records (EMR). All patients fulfilling the modified Duke’s criteria [8] for definite or possible IE were included in the study. The first admission during the study period was considered the index admission for patients with more than one episode of IE. Patients younger than 18 years were excluded.

### Data collection

Data regarding patient demographics, comorbidities and recent hospital admissions were documented. Healthcare-associated infective endocarditis (HCAI) was defined as IE in patients with health care contact before the index hospitalization. Health care contact constituted a history of receiving intravenous therapy (including chemotherapy), transfer from a specialized nursing care facility, haemodialysis, or hospitalization for at least 2 days in the 90 days prior to the index admission.

Presenting features and examination findings were recorded. Documented laboratory variables included haemoglobin (Hb), white blood cell count (WBC), platelet count, urine haemoglobin, serum creatinine, erythrocytes sedimentation rate (ESR), C-reactive protein (CRP), serum albumin and troponin I. Results on admission (i.e. baseline) and results after 2 weeks of IE treatment were analysed for all those parameters. Blood results at the time when the IE diagnosis was made were considered baseline results for those patients who developed IE during the admission.

Every patient had at least 4 blood samples taken for blood culture. Automated laboratory systems were utilised for pathogen identification and antimicrobial susceptibility testing. Genus and species of organisms cultured and their respective sensitivities were recorded. The type of echocardiographic modality [transthoracic echocardiography (TTE) vs. trans-oesophageal echocardiography (TOE)] and its results were documented. All available echocardiographic cinematic loops were reviewed by two consultant cardiologists for the presence of rheumatic heart disease (RHD) according to the 2012 WHF criteria for the echocardiographic diagnosis of RHD [9]. Type of antibiotics used for initial empirical treatment and subsequent definite treatment were documented. All patients included in this study completed 4 to 6 weeks of intravenous antibiotics. For all patients undergoing surgery; timing, type of surgery and outcome were recorded.

### Outcome measures and statistical analysis

The outcome was recorded as “death” or “discharged alive”. Results were analysed using SPSS for Windows version 24. Data are summarized as a percentage or mean ± standard deviation (SD). Categorical variables were compared using Chi-square or Fisher-Exact test as appropriate, while Student t-test was used for continuous variables. A p-value < 0.05 was considered significant.

## Results

### Recruitment

**Fig. 1:**
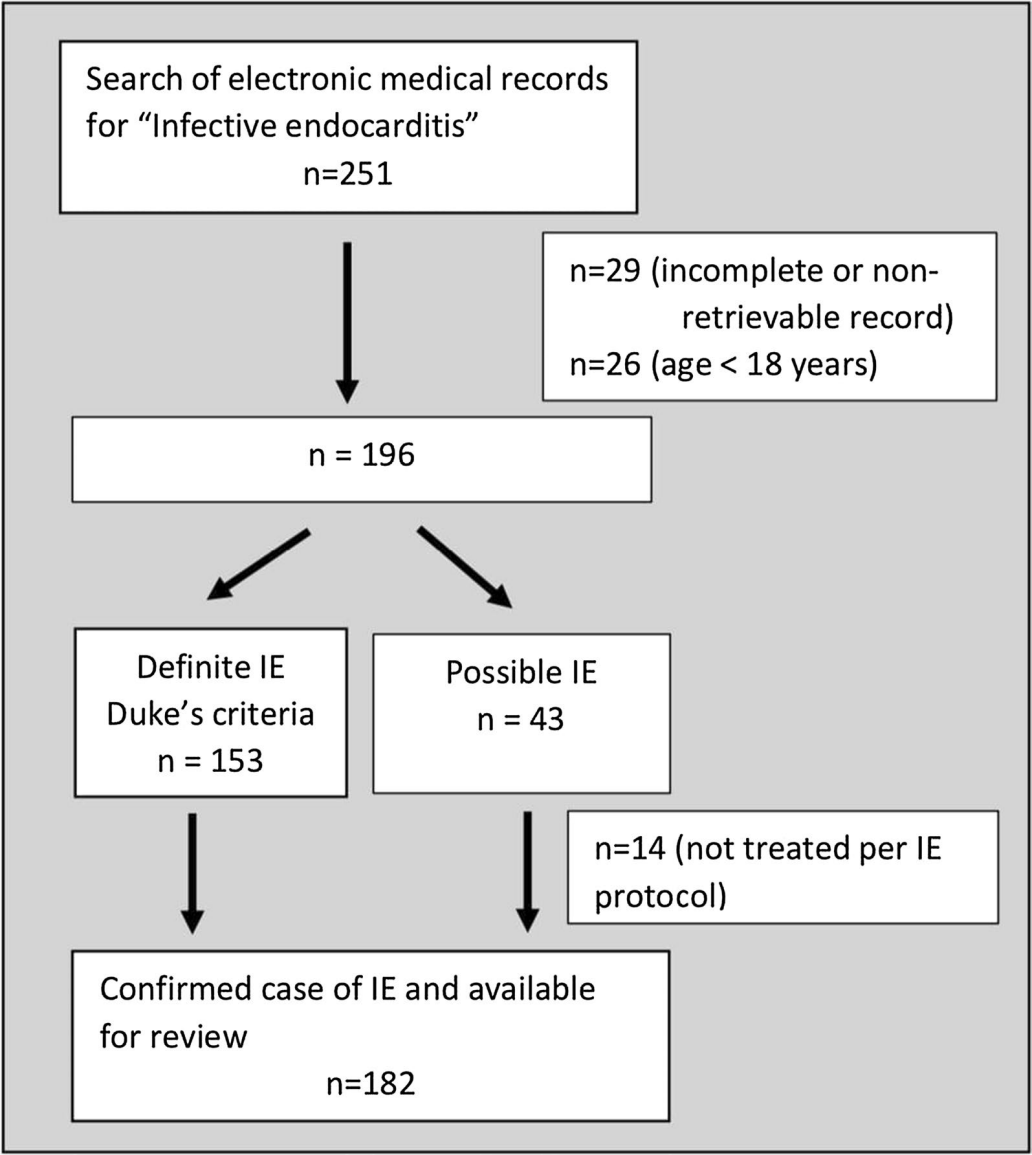
Patient recruitment

A search of the electronic medical records database and the admission logbooks yielded 251 patients with the label “IE”. The folders of 29 patients treated for IE between 2005 and 2007 were incomplete or could not be retrieved. Twenty-six patients were excluded as they were under 18 years of age. The details of the remaining 196 patients were analysed for the presence of the modified Dukes criteria for definite or possible IE [8]. The modified Duke’s criteria for definite IE were fulfilled by 153 patients which were consequently included in the study. Fourteen out of 43 patients who fulfilled the criteria for possible IE did not receive antibiotic treatment as per IE protocol and were excluded from the study. A total of 182 patients remained for analysis as shown in Fig. 1.

### Baseline demography

IE was more common in males [n = 128 (70%)] than in females [n = 54 (30%)]. The mean age was 50.5 ± 17.6 years. The mean duration of illness prior to admission was 20 (± 24) days. Eleven patients (6%) had a history of previous infective endocarditis. Common co-morbidities included diabetes (29.7%), hypertension (34.6%) and ischaemic heart disease (11.5%). Underlying immunosuppression was present in 14 (7.6%) patients. Intravenous drug abuse was reported by 9 (4.9%) patients. Seventeen patients (9.3%) were on regular haemodialysis while 11 (6%) patients had central venous catheters.

### Incidence

**Fig. 2:**
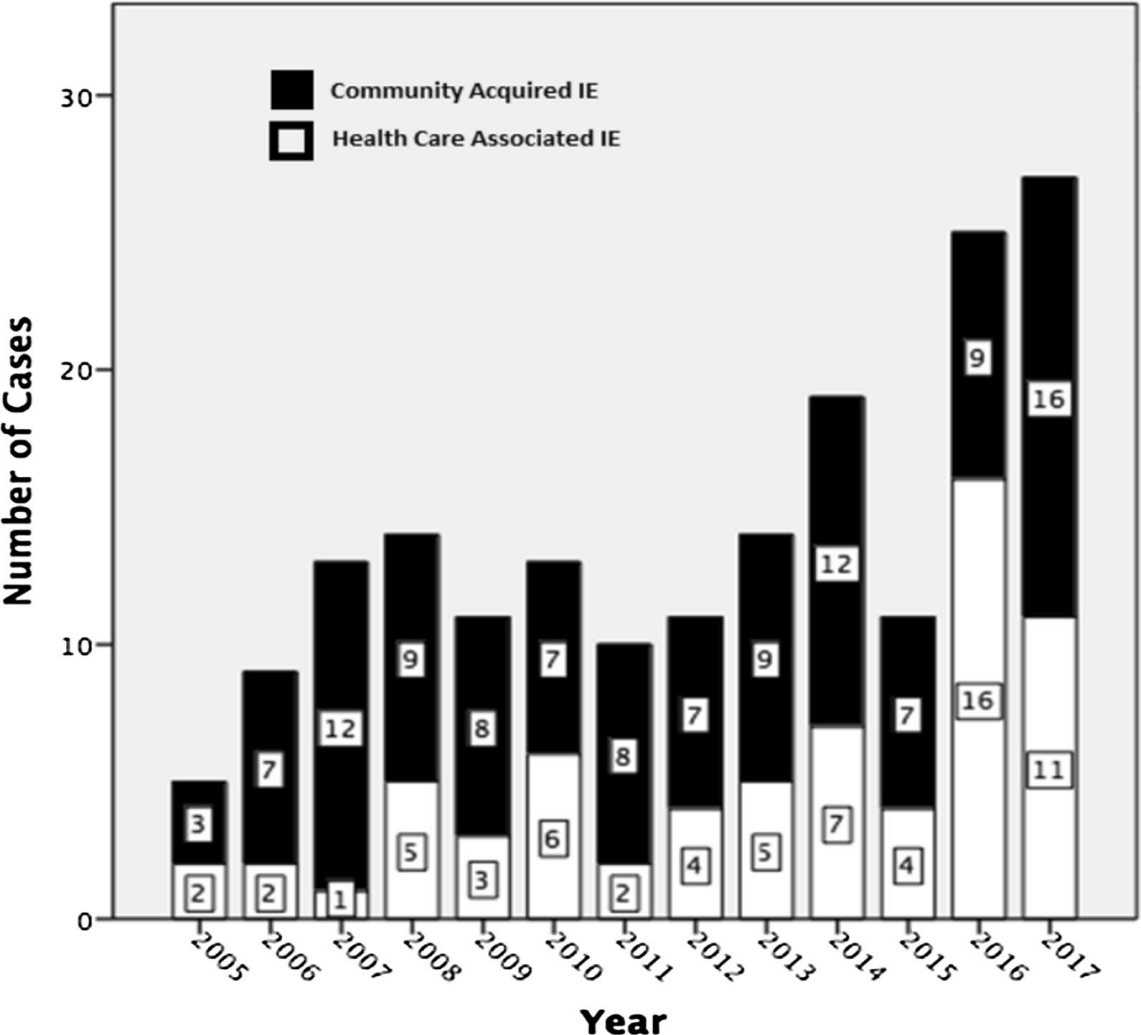
Infective Endocarditis incidence per year

Native valves were affected by the IE in 171 (94%) cases. Only 11 (6%) patients had prosthetic valve IE. Healthcare-associated IE (HCAIE) was recorded in 68 (37.4%) patients and community-acquired IE (CAIE) in 114 (62.6%) patients. The number of IE patients increased progressively over the 13-year study period with a particular increase over the last 6 years Fig. 2. IE admission rates almost tripled from 25/100,000 admissions in 2012 to 59/100,000 admissions in 2017. The mean incidence of IE was 30.7/100,000 adult admissions.

### Clinical presentation

Most patients 152 (83.5%) reported fever on admission. The mean duration of fever was 20 (± 24) days. The mean time to establish the diagnosis of IE was 4 (± 5 days). At least one major complication at the time of admission was documented in 59 (32.4%) patients. Complications comprised of heart failure in 38 (20.1%), systemic embolization in 21 (11.5%) and stroke in 10 (5.5%) patients. A murmur was present in 57 (31%) patients. Vascular and immunological evidence of IE was found in 48 (26.4%) and 13 (5.1%) patients respectively. Thirty-five (19.2%) patients had clinically splenomegaly and 24 (13.2%) hepatomegaly.

### Blood parameters

Anaemia was diagnosed on admission for 152 (68.7%) patients. The mean haemoglobin was 10.6 (± 2.4) g/dl and the mean white blood cell count was 13.8 (± 7.1). Nine (4.9%) patients had leucopenia. The mean platelet count on admission was 232 (± 135). Thrombocytopenia (< 150,000/ml) was present in 52 (28.6%) patients. Most patients 145 (79.7%) had hypoalbuminemia on admission (albumin < 35 g/dl). The mean erythrocyte sedimentation rate was 61 (± 33) and the mean CRP was 9.2 (± 6.7). Renal impairment was common with a mean creatinine of 158 (± 183) micromol/l. Raised Troponin I was found in 80 (44%) patients with a mean level of 0.45 (± 1.6).

### Echocardiographic features

All patients underwent transthoracic echocardiography (TTE) at least once, while transoesophageal echocardiography (TOE) was done for only 72 (39.6%) patients. TTE missed vegetations in 8 (4.4%) patients which were consequently identified by TOE. Left-sided IE was by far more common than right-sided IE [n = 159 (87.4%) vs. n = 18 (9.9%)]. Bilateral involvement was seen in 3 (1.6%) cases. Combinations of infected valves were seen in 11 (6.0%) cases. Concurrent IE affected most commonly the aortic and mitral valves [n = 8 (4.4%)].

Fourteen (7.7%) patients had underlying congenital heart disease comprising of atrial septal defects (n = 2), ventricular septal defects (n = 9) and cyanotic heart lesions (n = 3). Another 14 (7.7%) patients had prosthetic valve endocarditis. Echocardiographic cinematic loops for review were available for 120 patients. 50 (42%) of those fulfilled the WHO criteria for the presence of rheumatic heart disease (RHD).

### Microbiology data

Positive blood cultures were available for 142 (78%) patients. The mean duration to achieve culture clearance was 6 (± 5) days. Pathogens identified by blood culture in descending frequency were staphylococcus group [n = 58 (40.8%)], streptococcus group [n = 51 (35.9%)], *Enterococcus* species [n = 13 (9.2%)], HACEK [n = 11 (7.7%)], *Candida* sp. [n = 3 (2.1%)] and other bacteria [n = 6 (4.2%)]. *Staphylococcus aureus* infection was most common in the HCAIE group.

Among 58 *Staphylococcus* spp. isolated, 44 (75.8%) were methicillin-sensitive *S. aureus*, followed by 10 (17.2%) MRCoNS and 4 (6.9%) methicillin-resistant *S. aureus*. Almost all of the streptococcus species isolates were penicillin-sensitive [50 out of 51, 98%)]. A penicillin-resistant strain was found in only one patient (*Streptococcus mitis*—sensitive to ceftriaxone/vancomycin). All HACEK group organisms (n = 11) were sensitive to ceftriaxone, but only 3 (27.3%) of them were sensitive to ampicillin. Nine out of 13 (69.2%) *Enterococcus* species were ampicillin-sensitive, while 4 (30.8%) were ampicillin-resistant (sensitive to vancomycin). Microbial agents and respective resistance patterns are shown in Table 1.

**Table 1:** Microbial agents and resistance pattern of culture positive cases (n = 142)

Organism	n	%	Sensitive to	
			Methicillin	Vancomycin
*Staphylococcus* spp.	58	40.8		
*Staphylococcus aureus* spp.	48	33.8		
*MSSA*	44	31.0	44	–
*MRSA*	4	2.8	0	4
MRCoNS	10	7.0	0	10

			Penicillin	Vancomycin
Streptococcus spp.	51	35.9		
Viridans group	41	28.9	40	**–**
*Strep. mutans*	3	2.1	3	–
*Strep. godonii*	5	3.5	5	–
*Strep. oralis*	3	2.1	3	–
*Strep. mitis*	8	5.6	7	1
*Strep. sangunis*	6	4.2	6	–
*Strep. angiosus*	1	0.7	1	–
*Strep viridans*-*NOS*	15	10.6	15	–
Other streptococcus	10	7.0	10	–

			Ampicillin	Cephalosporin
HACEK group	11	7.7		
*Haemophilus parainfluenza*	5	3.5	2	5
*Haemophilus influenza*	1	0.7	1	1
*Haemophilus aphrophilus*	2	1.4	0	2
*Cardiobacterium* species	1	0.7	0	1
*Aggregatibacter* species	1	0.7	0	1
*Eikenella* species	1	0.7	0	1

			Ampicillin	Vancomycin
Enterococcus group	13	9.2		
*Enterococcus faecalis*	5	3.5	2	3
Other *Enterococcus* spp.	8	5.6	7	1
*Enterococcus* sp*. NOS*	6		6	0
*Enterococcus gallinarum*	1		1	–
*Enterobacter* sp.	1		Gentamycin/Ciprofloxacillin	1
Fungal	3	2.1		
*Candida parapsilosis*	1		Caspofungin	
*Aureobasidium* species	1		Amphotericin	
*Candida tropicalis*	1		Fluconazole, Voriconazole	
Other GPB	3	2.1		
*Propionibacterium acnes*	1		Penicillin, Ampicillin, Erythromycin.	
*Abiotrophia defectiva*	1		Penicillin, Cephalosporin.	
*Corynebacterium*	1		Vancomycin	
Other GNB	3	2.1		
*E. coli*	1		Ampicillin, Cephalosporin, Ciprofloxacin	
*Klebsiella pneumonia*	1		Penicillin, Cephalosporin	
*Providencia* sp.	1		Ampicillin, Cephalosporin, Gentamycin, Bactrim	

*MRCoNS* methicillin-resistant coagulase-negative staphylococcus, *NOS* not otherwise specified, *MSSA* methicillin-sensitive *Staphylococcus aureus*, *MRSA* methicillin-resistant *Staphylococcus aureus*, *NA* not applicable

### Antibiotic prescription

Empirical antibiotics were used while awaiting culture result. The majority of patients (65%) were treated with two empirical agents, typically comprising of a combination of gentamycin and benzylpenicillin or cloxacillin. Definitive therapy consisted of 6 weeks of culture-directed antibiotics for culture positive cases. 72 (39.6%) patients were treated with a single antibiotic while 110 (60.4%) patients received a combination of two antibiotics.

### Outcome and complications

The overall mortality rate was high. Sixty-five (35.7%) patients died in hospital, while 117 (64.3%) were discharged alive. The mean length of stay was 39.2 (± 21.4) days. Altogether, 182 patients had 7126 days of in-hospital treatment.

Surgery was performed for 58 (31.9%) patients, while 124 (68.1%) underwent medical therapy only. In-hospital surgery was performed for 36 (19.8%) patients, while 22 (12.1%) patients underwent surgery at a later stage post discharge.

Most patients [n = 163 (85.7%)] had at least one complication: 142 (78%) had valvular dysfunction, 52 (28.6%) patients developed heart failure, and 62 (34.1%) patients had systemic embolization. The embolic complications comprised of stroke [n = 35 (19.2%)], pulmonary infarction or abscess [n = 25 (13.7%)], splenic abscess or infarction [n = 6 (8.8%)]. Acute kidney injury occurred in 59 (32.4%) patients. Electrocardiographic abnormalities were documented in 30 (16.5%) patients and included atrial fibrillation [n = 17 (9.3%)], supraventricular tachycardia [n = 8 (4.4%)], ventricular tachycardia (n = 3) and first-degree AV block (n = 2).

## Discussion

### Evolving epidemiology

Literature from developed health care systems has been showing an epidemiological evolution of IE over the past decade Table 2. There is a lack of IE epidemiological data from developing countries, thus preventing conclusions regarding local IE trends.

**Table 2:** Major IE studies

	This study	Ibrahim et al. [19]	Gupta et al. [15]	Xu et al. [10]	Poesen et al. [20]	Watt et al. [6]	Toyoda et al. [2]
Time of study	2005–2017	2012–2013	2005–2010	2008–2015	2003–2010	2010–2012	1998–2013
Country	Malaysia	Malaysia	India	China	Belgium	Thailand	US
Episodes	182	50	61	174	88	132	75,829
Incidence (per 100,000 adm)	31	NA	80	NA	54	NA	7.6–9.3
Age (years) (SD/IQR)	51 ± 17.62	42 ± 16.4	49 ± 13.7	48 ± 15.7	72 IQR 59–81	47 IQR 16–85	62 ± 18.9
Male: female ratio	2.4:1	2.8:1	3.3:1	1.9:1	2:1	2.2:1	1.4:1
Predispositions (%)							
RHD	42	NA	37.7	14.9	2.3	28.0	NA
CHD	7.7	6.0	22.9	29.9	5.7	8.3	4.5
Dialysis	9.3	6.0	NA	3.4	4.5	NA	18.4
IVDU	4.9	26.0	1.6	0.6	1.1	NA	12.5
HIV	2.7	4.0	NA	NA	NA	NA	1.7
Culture positive (%)	78	68	67.2	60.3	89.8	45.5	73.0
HCAIE (%)	37.4	NA	NA	NA	NA	NA	52.9
PVE (%)	6	NA	31	5.2	8	9.9	12.9
Organisms (%)							
Streptococcus	35.9	20	21.4	37.4	44.4	22	26.6
Staphylococcus	40.8	36	21.4	13.8	21.7	5.3	38.5
Complications (%)							
CHF	28.6	NA	47.5	69.0	51.1	48	NA
Stroke	19.2	10.0	11.5	16.1	NA	NA	NA
Surgery for IE (%)	32	20.0	49.2	43.7	29.5	75.8	NA
In-hospital death (%)	36.7	28.0	6.6	10.9	17.0	11.4	24.0^a^

*SD* standard deviation, *IQR* interquartile range, *RHD* rheumatic heart disease, *CHD* congenital heart disease, *IVDU* intravenous drug use, *HCAIE* health care associated IE, *PVE* prosthetic valve IE, *CHF* congestive heart failure, *IE* infective endocarditis, *NA* not available

^a^90 days mortality in this study

We found an incidence of 31/100,000 IE cases per adult admissions over the entire study period which appears to be higher than in developed countries. In addition, we noted an increasing trend in the IE incidence. There was an almost threefold increase in the incidence rate from 25/100,000 to 59/100,000 over the last 6 years of the study period. Our patient cohort appears to be younger (50.4 ± 17 years) than the IE population of western countries where patients are typically in their 60ies or 70ies. This finding is in keeping with that of other Asian studies conducted in China [10] (mean age 47.8 ± 15.7 years) and Thailand [6] (mean age 47 years, IQR 16–85 years) and likely reflects the overall younger populations of developing nations. Despite this, patients in our study had a high rate of non-communicable diseases like diabetes (29.7%), hypertension (34.6%) and ischaemic heart disease (11.5%). The HCAIE rate was high in our centre (37.4%). A trend towards increasing HCAIE has been observed in many western tertiary referral centres. Toyoda [2] reported a HCAIE rate of 52.9% based on a large American IE database analysis with increased IE mortality in this group of patients. Fowler found that HCAIE is commonly associated with *S. aureus* infections, particularly methicillin-resistant *S. aureus* (MRSA) [1]. Our study identified staphylococcus predominance (40.8% staphylococcus vs 35.9% streptococcus). This is in contrast to regional studies done in China and Thailand that demonstrated still streptococcal predominance in their respective cohorts [6, 10]. Viridans group streptococci account for only 17–26% of IE in western populations where RHD is now disappearing [11]. Staphylococcus (31–54%) is the most common culprit organism in high-income countries, with methicillin-sensitive *S. aureus* (MSSA) as the most common agent in native valve infective endocarditis (NVIE) and methicillin-resistant *S. aureus* (MRSA) for health care associated infective endocarditis (HCAIE) [11]. MRSA has also overtaken coagulase-negative staphylococci as the main cause of PVIE in recent studies [1, 12]. Higher staphylococcus IE incidence is typically reported in referral centres and has been shown to correlate with higher mortality [13, 14].

The prevalence of RHD in our IE patients (42%) is still much higher than in Western populations where degenerative valvular pathology and valvular prostheses [1, 5] constitute the main underlying pathological substrates. The culture-negative IE rate (22%) in our centre is comparable with those reported in developed health care systems (about 10–20%). Relevant serological investigations for *Coxiella burnetii*, *Legionella* spp., *Brucella* spp., *Bartonella* spp., and *Chlamydiae* spp. should be performed where epidemiologically applicable [4, 11]. Much higher rates of culture-negative IE are commonly reported in regional studies: 30–40% in China and India; 65% in Thailand [6, 10, 15]. Often, only limited investigations can be carried out due to financial constraints when a more extensive workup would be required to accurately identify the fastidious organism in developing countries.

Echocardiography demonstrated vegetations in 99.5% of cases diagnosed as IE in our study. This high rate is most likely due to improved diagnostic imaging modalities [10]. However, only 39.6% of the patients had a TOE done. This contrasts with reports from developed healthcare systems where the majority of patients undergo TOE to detect complications early. In our study, TTE missed vegetation in 8 (4.4%) patients emphasizing the diagnostic importance of TOE in suspected IE. The mitral valve was the most commonly affected valve.

### IE mortality and surgery

The in-hospital mortality rate of 36.7% in our study population was high. Recent studies conducted in developed health care systems reported persistently high mortality rates of 20–22%, 30% and 45% for the in-hospital period, at 1 year and at 5 years respectively [7, 12, 16, 17]. The root cause for the lack of improvement in IE mortality in developed health care systems is believed to be the increasing numbers of older and immunocompromised patients with HCAIE with more resistant organism [1, 4, 5, 11]. The same trend seems to apply to urban tertiary centres of developing countries: increasing rates of HCAIE in a multimorbid, older population resulting in persistently high mortality rates despite optimal medical care.

Early surgery has been shown in RCTs to significantly reduce in-hospital death and embolic complications [16, 18]. In developed medical systems, about 40–50% of patients are managed surgically whereas the surgical intervention rate in our study was only 32% and might partially explain the rather high mortality. Mortality rates reported in other developing countries, such as China (11%) [10] and Thailand (11.4%) [6] are much lower than local figures and are associated with higher surgery rates (44% and 75% resp.).

### Study limitations

As a retrospective study, this study has limitations associated with retrospective data collection. 29 patients identified as IE cases had to be excluded as the medical records could not be retrieved. The data may not be generalizable as the hospital is a tertiary referral centre with inherent selection and referral bias. There are strong urban/rural socio-economic disparities in developing nations with regards to standard of care and health care access. Prospective studies with larger sample sizes are needed to confirm our findings.

## Conclusions

Staphylococcus is the new etiologic champion, likely reflecting the transition of the healthcare system to a more developed state entailing more invasive procedures and serving a generally older and more multi-morbid population with increasing HCAIE rates. Despite the epidemiological transition to staphylococcus predominance, RHD remains as an important predisposing factor in our local IE population. Streptococcus viridans is still an important etiological agent of IE. The incidence rate of IE is increasing.

## Data Availability

The datasets used and/or analysed during the current study are available from the corresponding author on reasonable request.

## References

[1] Fowler VG, Miro JM, Hoen B, Cabell CH, Abrutyn E, Rubinstein E, Corey GR, Spelman D, Bradley SF, Barsic B, Pappas PA (2005). *Staphylococcus aureus* endocarditis: a consequence of medical progress. JAMA.

[2] Toyoda N, Chikwe J, Itagaki S, Gelijns AC, Adams DH, Egorova NN (2017). Trends in infective endocarditis in California and New York State, 1998–2013. JAMA.

[3] Hoen B, Alla F, Selton-Suty C, Béguinot I, Bouvet A, Briançon S, Casalta JP, Danchin N, Delahaye F, Etienne J, Le Moing V (2002). Changing profile of infective endocarditis: results of a 1-year survey in France. JAMA.

[4] Cahill TJ, Prendergast BD (2016). Infective endocarditis. Lancet.

[5] Hoen B (2006). Epidemiology and antibiotic treatment of infective endocarditis: an update. Heart.

[6] Watt G, Pachirat O, Baggett HC, Maloney SA, Lulitanond V, Raoult D, Bhengsri S, Thamthitiwat S, Paupairoj A, Kosoy M, Ud-Ai N (2014). Infective endocarditis in northeastern Thailand. Emerg Infect Dis.

[7] Abdulhak AA, Baddour LM, Erwin PJ, Hoen B, Chu VH, Mensah GA, Tleyjeh IM (2014). Global and regional burden of infective endocarditis, 1990–2010: a systematic review of the literature. Glob Heart.

[8] Li JS, Sexton DJ, Mick N, Nettles R, Fowler VG, Ryan T, Bashore T, Corey GR (2000). Proposed modifications to the Duke criteria for the diagnosis of infective endocarditis. Clin Infect Dis.

[9] Reményi B, Wilson N, Steer A, Ferreira B, Kado J, Kumar K, Lawrenson J, Maguire G, Marijon E, Mirabel M, Mocumbi AO (2012). World Heart Federation criteria for echocardiographic diagnosis of rheumatic heart disease—an evidence-based guideline. Nat Rev Cardiol.

[10] Xu H, Cai S, Dai H (2016). Characteristics of infective endocarditis in a tertiary hospital in East China. PLoS ONE.

[11] Martinez G, Valchanov K (2012). Infective endocarditis. Continuing education in anaesthesia. Crit Care Pain.

[12] Chambers J, Sandoe J, Ray S, Prendergast B, Taggart D, Westaby S, Arden C, Grothier L, Wilson J, Campbell B, Gohlke-Bärwolf C (2014). The infective endocarditis team: recommendations from an international working group. Heart.

[13] Bergin SP, Holland TL, Fowler VG, Tong SY (2017). Bacteremia, sepsis, and infective endocarditis associated with *Staphylococcus aureus*. Curr Top Microbiol Immunol.

[14] Ben-Ami R, Giladi M, Carmeli Y, Orni-Wasserlauf R, Siegman-Igra Y (2004). Hospital-acquired infective endocarditis: should the definition be broadened?. Clin Infect Dis.

[15] Gupta A, Kaul U, Varma A (2013). Infective endocarditis in an Indian setup: are we entering the ‘modern’era?. Indian J Crit Care Med.

[16] Kang DH, Kim YJ, Kim SH, Sun BJ, Kim DH, Yun SC, Song JM, Choo SJ, Chung CH, Song JK, Lee JW (2012). Early surgery versus conventional treatment for infective endocarditis. N Engl J Med.

[17] Prendergast BD (2006). The changing face of infective endocarditis. Heart.

[18] Netzer RO, Zollinger E, Seiler C, Cerny A (2000). Infective endocarditis: clinical spectrum, presentation and outcome. An analysis of 212 cases 1980–1995. Heart..

[19] Ibrahim KS, Ismail JR, Yusof Y, Yuhana Y, Saman MS, Khir NR, Lim CW, Ibrahim OZ, Rahman EA, Chua N, Abidin HA (2017). Pattern and predictors of outcomes for infective endocarditis in North Kuala Lumpur. Heart India..

[20] Poesen K, Pottel H, Colaert J, Niel CD (2014). Epidemiology of infective endocarditis in a large Belgian non-referral hospital. Acta Clin Belg.

